# Dexamethasone Down-Regulates the Expression of microRNA-155 in the Livers of Septic Mice

**DOI:** 10.1371/journal.pone.0080547

**Published:** 2013-11-11

**Authors:** Zhong-hua Wang, Yan-bing Liang, Hao Tang, Zhi-bin Chen, Zhen-yu Li, Xu-chu Hu, Zhong-fu Ma

**Affiliations:** 1 Department of Intensive Care Unit, Guangdong General Hospital, Guangzhou, Guangdong, China; 2 Department of General Internal Medicine, First Affiliated Hospital of Sun Yat-sen University, Guangzhou, Guangdong, China; 3 Department of Parasitology, Zhongshan School of Medicine, Sun Yat-sen University, Guangzhou, Guangdong, China; University of North Dakota, United States of America

## Abstract

To investigate the expression of microRNA-155 (*miRNA-155*) in the livers of mice with lipopolysaccharide (LPS)-induced sepsis and to determine the role of dexamethasone (DXM) in the regulation of *miRNA-155* expression, we pretreated mice with or without DXM prior to LPS exposure. Our study demonstrated that the expression of *miRNA-155* and inflammatory factors increased in the liver tissues of mice with LPS-induced sepsis and that DXM down-regulated their expression in a dose-dependent manner. Moreover, DXM alone inhibited the expression of *miRNA-155* to below the baseline level, but did not impact the expression of inflammatory factors, suggesting that the down-regulation of *miRNA-155* by DXM may partially, but not completely, depend on the suppression of pro-inflammatory cytokines by DXM. Our data indicate that the overexpression of *miRNA-155* in the livers of mice with LPS-induced sepsis may play an important role in the pathological processes of sepsis and that the down-regulation of *miRNA-155* by DXM may be a novel mechanism regulating inflammation and immunity.

## Introduction

Sepsis, an infection-induced systemic inflammatory response syndrome, remains as the main cause of death in critical patients and the process is complex. Several regulatory mechanisms are involved in the development of sepsis. The discovery of microRNAs (miRNAs) suggests a novel regulatory mechanism for this process. 

miRNAs are a class of small non-coding RNA molecules with critical roles in cell proliferation, differentiation, and apoptosis [1]. Ample evidence suggests that miRNAs are key regulators in animal development and are involved in a variety of human diseases [2]. The transcription of primary miRNAs (pri-miRNAs) by RNA polymerase II is the first step of miRNA biogenesis. In the nucleus, pri-miRNAs are converted into hairpin precursor miRNAs (pre-miRNAs). Pre-miRNAs are transported into the cytoplasm where they are further processed into mature miRNAs consisting of 22 nucleotides; Dicer is involved in these final processing events. Mature miRNAs have the ability to bind to the 3’ untranslated region (UTR) of their target mRNAs in a sequence-specific manner [3], initiate partial or full degradation of mRNA transcripts, and regulate the expression of protein-coding genes at the post-transcriptional level. The effects of miRNAs are mediated by inhibiting the translation or degradation of the target mRNA. TNF-α, an important inflammatory factor, is inhibited indirectly by *miRNA-146a*, which targets TRAF6 and IRAK1 [4], and directly by *miRNA-125b*, which targets the 3’UTR of *TNF-α* mRNA[5]. More than 700 miRNAs have been identified in mammals to date. These miRNAs are associated with diverse biological processes, such as the regulation of insulin secretion, viral infection, and tumorigenesis. Previous studies have indicated that most miRNAs play a role in innate immune responses and inflammation. In response to inflammation, some miRNAs are up-regulated, while some others are down-regulated. For example, the expression of *miRNA-146a*, *miRNA-155*and *miRNA-21* are up-regulated in monocytes challenged by LPS [4,6,7], whereas that of *miRNA-125b* is down-regulated [5].

*miRNA-155* was initially discovered as a proto-oncogene in lymphoma [8]. The overexpression of *miRNA-155* has been detected in B-cell lymphomas[9][10] and chronic lymphocytic leukemia[11]. It is also overexpressed in various solid tumors, including lung[12], breast[13], pancreatic[14], and thyroid cancers[15]. The roles of *miRNA-155* in various physiological and pathological processes, such as hematopoietic lineage differentiation, inflammation and immunity, have been identified recently [16]. *miRNA-155* is required for the development of T cells, B cells, and dendritic cells. Previous studies have shown that *miRNA-155* plays an important role in immunoglobulin class switching to IgG in B cells via the targeted repression of the transcription factor PU.1 [17] and activation-induced cytidine deaminase (AID) [18]. Other validated target genes of *miRNA-155*, such as interleukin-1 (IL-1), IkappaB kinase ε (*IKKε*), *Ets-1*, and *Meis1*, are associated with the hematopoietic and immune systems [19–21]. *miRNA-155* gene knockout mice displayed severe immune response deficiencies after pathogen exposure. In antigen-speciﬁc inﬂammatory responses against autologous tissue, *miRNA-155* has been shown to promote autoimmune inflammation [22]. Furthermore, *miRNA-155* has been found to be up-regulated in macrophages following stimulation by a broad range of inflammatory mediators [6]. LPS induces the expression of *miRNA-155* in the spleens of mice [5], but this increase in expression is not observed in other organs. The LPS-induced expression of *miRNA-155* should be investigated in the liver because this organ is commonly damaged in mice with sepsis.

Dexamethasone (DXM), a potent synthetic member of the glucocorticoid family, has anti-inflammatory, anti-allergic, anti-shock, and anti-endotoxin effects. DXM has been widely used to treat inflammatory and autoimmune diseases, including severe sepsis, multiple sclerosis, rheumatoid arthritis, asthma, and systemic lupus erythematosus. In the cytoplasm, DXM interacts with the glucocorticoid receptor and forms a ligand-receptor complex, which subsequently translocates to the nucleus. In the nucleus, the complex binds to genomic DNA and regulates the expression of both anti-inflammatory and inflammatory genes at the transcriptional level. Moreover, DXM may impact the activities of signal-dependent transcription factors, including members of the activator protein 1 (AP-1) and nuclear factor-κB (NF-κB) families [23], to negatively regulate inflammatory responses. To elucidate the mechanism of DXM in regulating the inflammatory and immune response in mice with sepsis, we established a mouse model of sepsis and determined the effect of DXM on the expression of *miRNA-155* in the liver.

## Materials and Methods

### Animals and treatment

Animal experiments were performed in strict accordance with the Guide for the Care and Use of Laboratory Animals of Sun Yat-sen University. The protocol was approved by the Committee on the Ethics of Animal Experiments of the First Affiliated Hospital of Sun Yat-sen University. A total of 350 female BALB/c mice (6-8 weeks of age and weighted 20-25 g) were purchased from the Experimental Animal Center of Sun Yat-sen University (Guangzhou, China). Mice were housed in a pathogen-free animal facility that was maintained at 24°C, 55% humidity, and had a 12 h light/dark cycle. All mice had free access to food and water, and they received humane care in accordance with the National Institutes of Health guidelines and the legal requirements of China. The mouse model of sepsis was developed by intraperitoneal injections of LPS as previously described[24]. 

Three hundred mice were divided randomly into five groups, with 60 mice in each group: <1> the normal saline (NS) control group received an intraperitoneal injection of 50 μl of NS; <2> the LPS group received an intraperitoneal injection of 15 mg/kg *E. coli* LPS (*Escherichia coli O55:B5*, Sigma, St. Louis, MO, USA); <3> the NS-LPS group received an intraperitoneal injection of 50 μl NS 1 hour prior to LPS exposure; <4> the DXM-LPS group received an intraperitoneal injection of 5 mg/kg DXM (Sigma, St. Louis, MO, USA) dissolved in 50 μl NS 1 hour prior to LPS exposure; and <5> the DXM group received an intraperitoneal injection of 5 mg/kg DXM dissolved in 50 μl NS. We prepared the DXM according to the manufacturer’s instruction. The LPS and DXM were dissolved in sterile saline solution under aseptic condition before injection. Ten mice from each group were killed at each indicated time point following LPS treatment (0, 2, 6, 12, 24, and 48 hours), and the liver tissue and peripheral blood were collected. 

The remaining 50 mice were divided equally into five groups which received injection of 0, 0.5, 2, 5, and 10 mg/kg DXM, respectively, dissolved in 50 μl NS 1 hour prior to LPS exposure and were killed at 12 h following LPS treatment. The liver tissues were collected.

### Measurements of serum ALT, TNF-α, IL-6, and IL-10 levels

The serum levels of alanine aminotransferase (ALT), TNF-α, IL-6, and IL-10 were measured. Blood samples from the NS and LPS groups were centrifuged at 1,000 ×*g* at 4°C for 20 minutes. Serum ALT was determined using a commercial kit (Wako Pure Chemical Industries, Ltd., Osaka, Japan). Serum levels of TNF-α, IL-10, and IL-6 were quantified using an ELISA kit (R&D Systems, Inc, Minneapolis, USA). Initially, the serum from each mouse was added to 96-well plates (10 μl/well) containing capture antibodies specific to mouse TNF-α, IL-6, and IL-10. Next, 100 μl of HRP-conjugated reagent was added to each well. The plates were incubated at 37°C for 1 hour, washed, and a substrate solution was added to each well. The plates were incubated for 15 minutes at 37°C, and subsequently, a stop solution was added to each well. Finally, the plates were read at 450 nm.

### Measurement of tissue TNF-α, IL-6 and IL-10 levels

Liver tissues were homogenized in ice-cold sterile saline at a ratio of 1 ml per 100 mg tissue using an Ultra-turrax T18 homogenizer (IKA, Germany ). The homogenates were centrifuged at 1,000 ×*g* at 4°C for 15 minutes. The supernatant was transferred to a new tube and stored at -80°C. The amount of total protein was detected using the Bradford reagent (Sigma, St. Louis, MO, USA) according to the manufacturer's instructions. A total of 50 μg of protein was used to examine tissue TNF-α, IL-6, and IL-10 levels with an ELISA Kit (R&D Systems, Inc, Minneapolis, USA).

### Histopathologic evaluation

The liver tissues were subjected to histological examination. All liver tissues were immersed in 4% formaldehyde and embedded in paraffin. The paraffin-embedded samples were then cut into 4 μm thick sections, deparaffinized with xylene, rehydrated through a series of decreasing concentrations of ethanol, and stained with hematoxylin-eosin (HE). All samples were examined and photographed using an OLYMPUS BX51WI optical microscope (Olympus, Japan).

### Extraction and measurement of miRNA

Total RNA was extracted from 100 mg of liver tissue with Trizol reagent according to the manufacturer’s protocol (Invitrogen, California, USA). A total of 2 μg RNA was used to synthesize first-strand cDNA using the One Step PrimeScript miRNA cDNA Synthesis Kit (Takara, Japan). The *miRNA-155* level was quantified by real-time PCR (qRT-PCR) using SYBR Premix Ex Taq II (Takara, Japan) with U6 small nuclear RNA as the internal normalized reference. Real-time PCR primers were provided by RiboBio (Guangzhou, China). The expression of *miRNA-155* was measured using the Roche Light Cycler 480 Real-Time PCR system. PCR reactions were performed at 95°C for 10 minutes followed by 40 cycles of denaturing at 95°C for 15 seconds and an annealing/extension at 60°C for 60 seconds. All reactions were performed in triplicate. The qRT-PCR data were analyzed and expressed as relative miRNA levels of the cycle threshold value, which was subsequently converted to fold change with 2^–△△CT^ method[25] .

### Statistical analysis

Statistical changes in cytokine and ALT expression were determined with the two-tailed student *T* test; the changes of miRNA expression were analyzed by analysis of variance (ANOVA). Differences were considered significant at *P* < 0.05.

## Results

### An acute inflammatory response was induced in mice with LPS-induced sepsis

**Figure 1:**
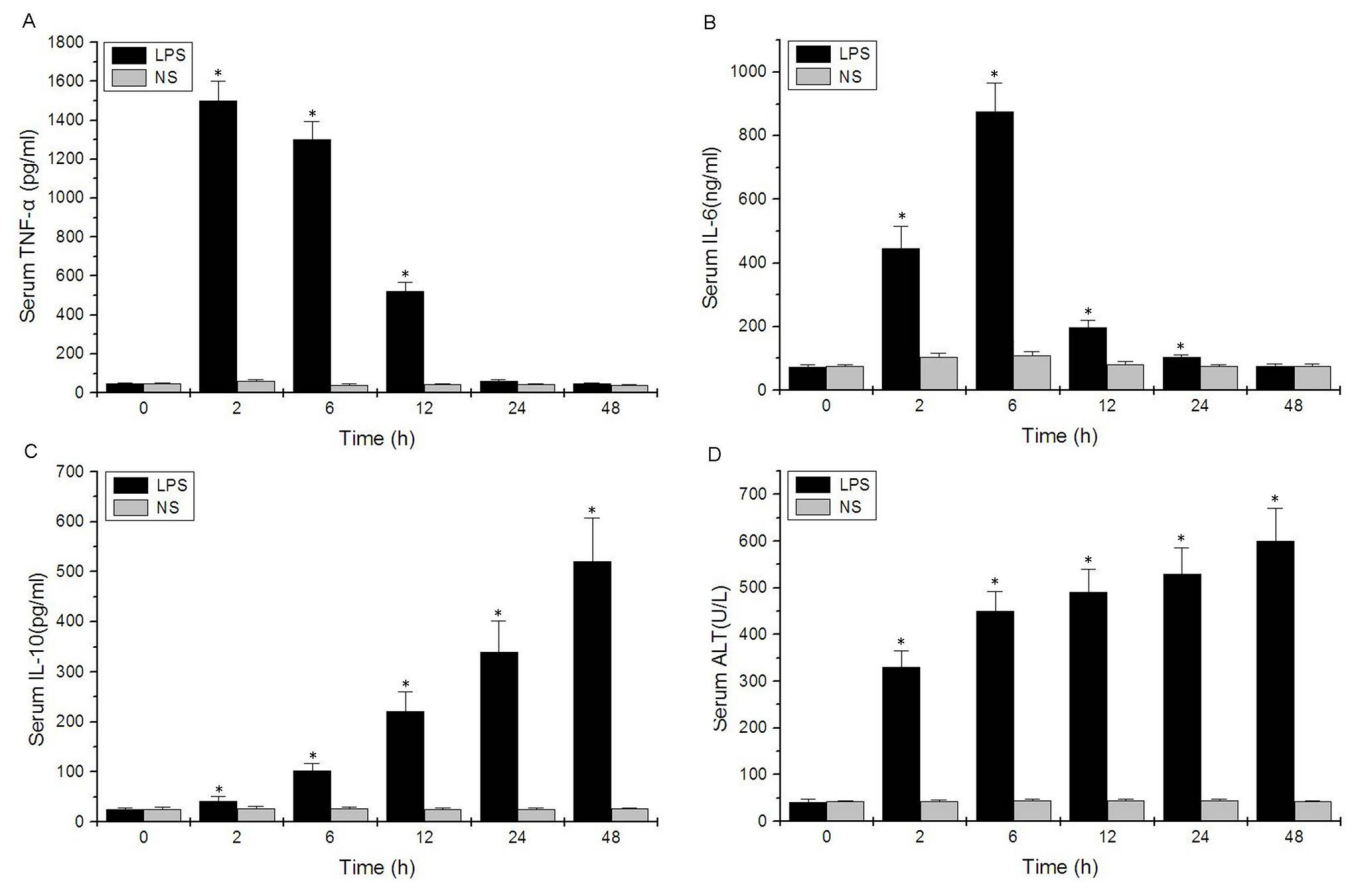
Serum levels of inflammatory cytokines and alanine aminotransferase (ALT) increase in mice with sepsis induced by lipopolysaccharide (LPS). (**A**) tumor necrosis factor-α (TNF-α), (**B**) interleukin-6 (IL-6), (**C**) IL-10 and (**D**) ALT. Data are provided as the mean ± standard deviation (SD) of 10 mice in each group (*P < 0.05 versus the NS group).

**Figure 2:**
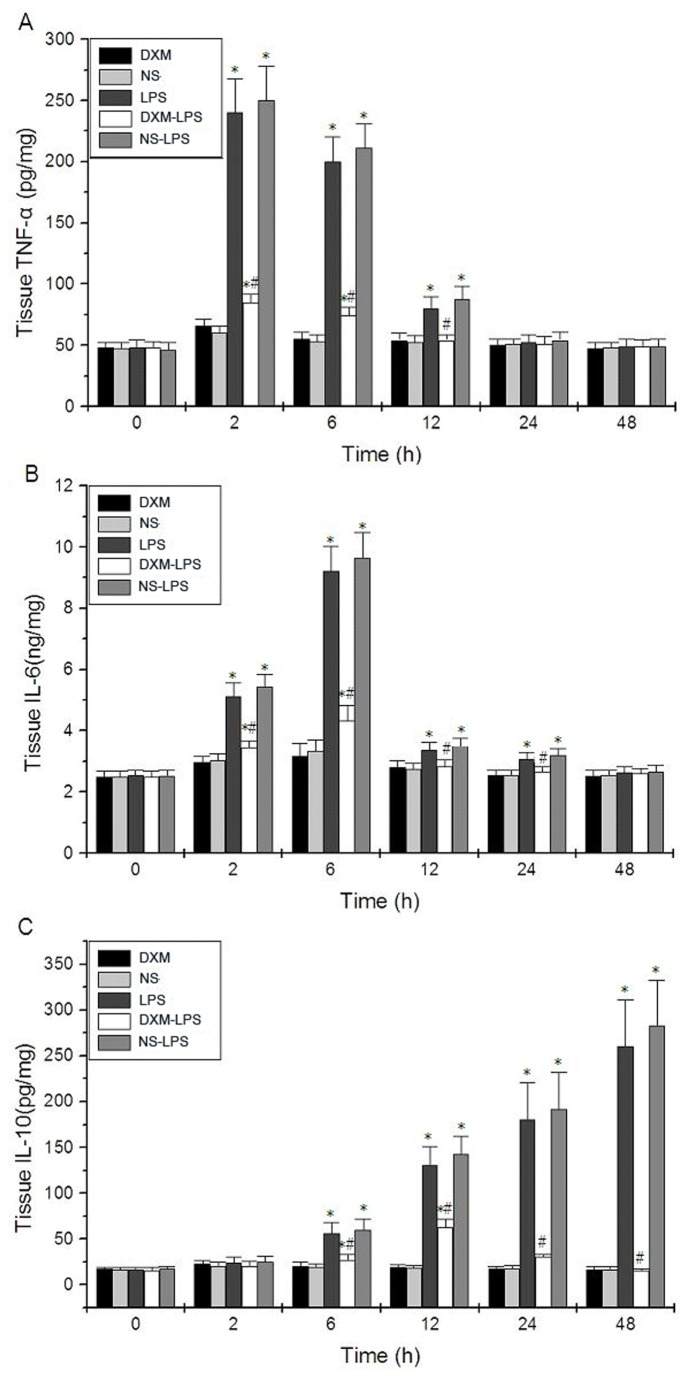
The levels of inflammatory cytokines in the livers of mice with LPS-induced sepsis **are suppressed by dexamethasone (DXM)**. Mice were divided randomly into five groups: NS group received an intraperitoneal injection of 50 μl of NS; LPS group received an intraperitoneal injection of 15 mg/kg LPS; NS-LPS group received an intraperitoneal injection of 50 μl NS 1 hour prior to LPS exposure; DXM-LPS group received an intraperitoneal injection of 5 mg/kg DXM dissolved in 50 μl NS 1 hour prior to LPS exposure; DXM group received an intraperitoneal injection of 5 mg/kg DXM dissolved in 50 μl NS. (**A**) TNF-α, (**B**) IL-6 and (**C**) IL-10. Data are provided as the mean ± SD of 10 mice in each group (* *P* < 0.05 versus the NS group; # *P* <0.05 for the DXM-LPS group versus the NS-LPS group).

After LPS exposure, the expression of inflammatory cytokines TNF-α, IL-6, and IL-10 were dramatically increased in serum (Figure 1A-C) and liver tissues of BALB/c mice (Figure 2A-C). Serum and tissue TNF-α levels simultaneously peaked at approximately 2 hours after LPS exposure and quickly declined to baseline levels over 12 hours (Figure 1A and 2A). Serum and tissue IL-6 levels peaked at 6 hours and declined by 12 hours, but IL-6 could still be detected at 24 hours after LPS exposure (Figure 1B and 2B). Serum and tissue IL-10 levels as well as serum ALT level increased with time after LPS injection (Figure 1C, 2C, and 1D). 

**Figure 3:**
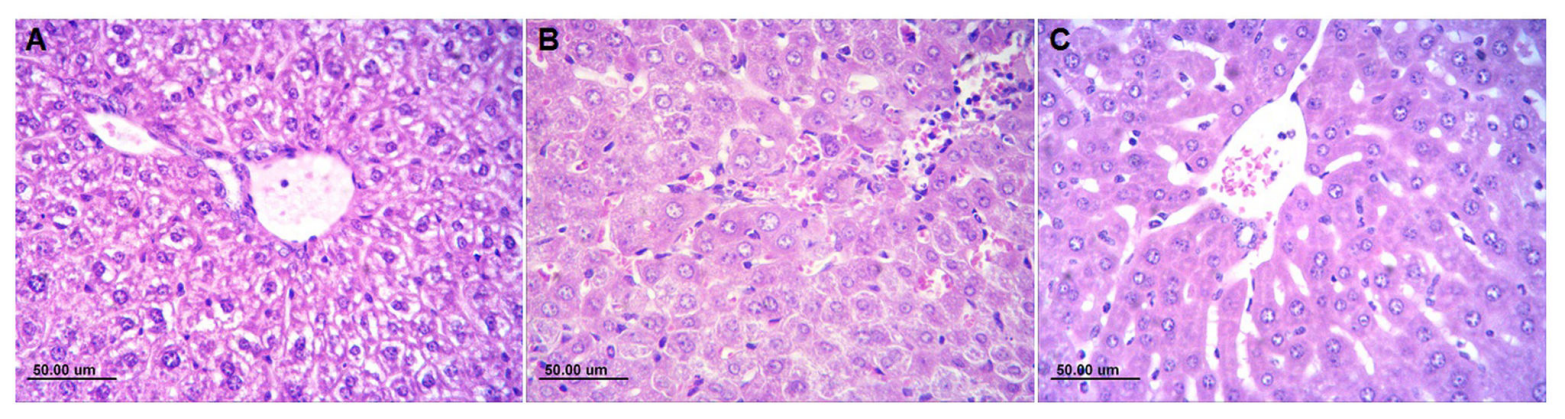
Dexamethasone (DXM) inhibits inflammation in mice with LPS-induced sepsis. **The liver tissues was examined pathologically at 12 hours after LPS exposure**. (**A**) the NS group : the liver tissue pathology is normal; (**B**)the LPS group: A large number of leukocytes infiltrated in the portal tracts and sinusoids. The hepatocyte vacuolation, architectural distortion, and nodular necrosis were evident; (**C**) the DXM-LPS group: a little number of leukocytes infiltrated in the portal tracts and sinusoids. Mild damage to liver cells.

Liver tissues were observed under a microscope with HE staining to investigate the inflammatory reaction. Compare to the normal liver tissue pathology (Figure 3A), The recruitment of inflammatory cells into the liver was observed 2 hours after LPS injection. A large number of leukocytes infiltrated in the portal tracts and sinusoids. Furthermore, hepatocyte vacuolation, architectural distortion, and nodular necrosis were evident at 12 hours after LPS injection (Figure 3B).

### Expression of *miRNA-155* was up-regulated in the livers of mice with LPS-induced sepsis

**Figure 4:**
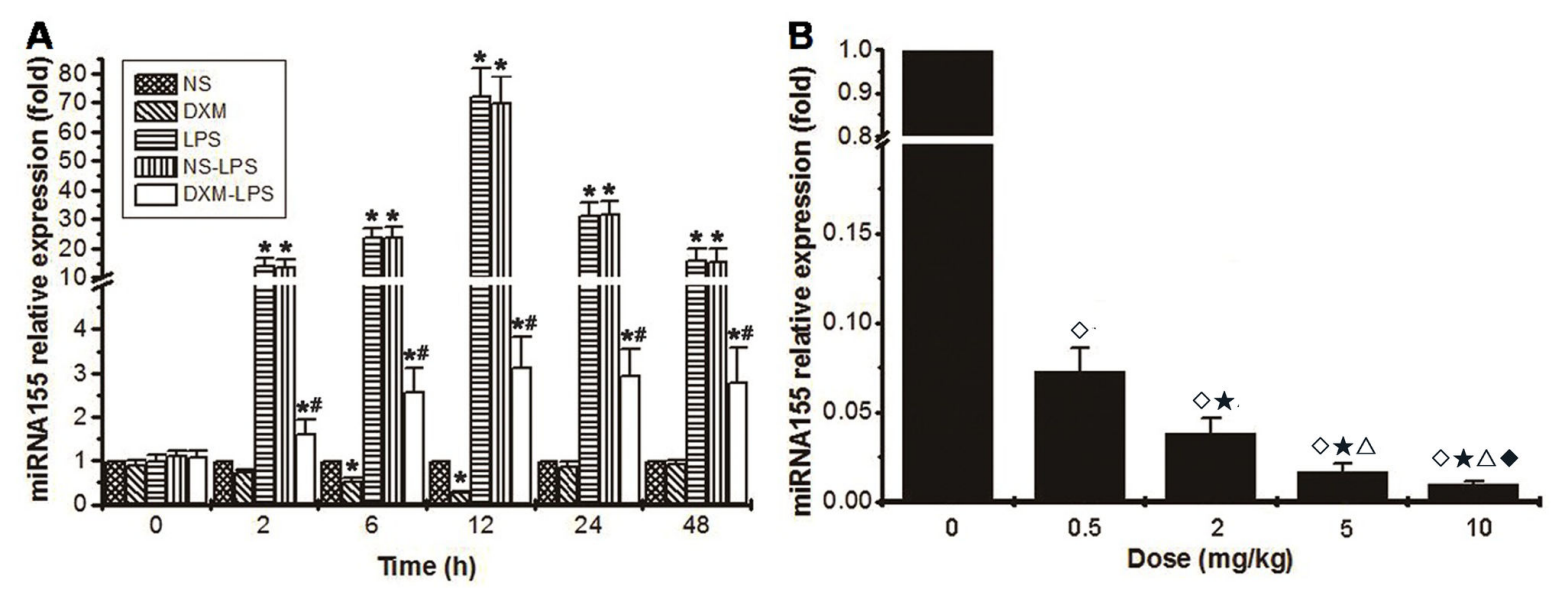
The expression of *miRNA-155* in the liver tissue of mice with LPS-induced sepsis was reduced by DXM. (**A**) the expression of *miRNA-155* in the liver tissues was detected by real-time polymerase chain reaction (RT-PCR). All values are presented as mean ± SD of 10 mice in each group. At each indicated time point, the *miRNA-155* level in the NS group was used as a reference (equivalent to 1) to calculate the relative expression of *miRNA-155* in other groups (*P < 0.05, versus NS group; # *P* <0.05, DXM-LPS group versus NS-LPS group). (**B**) DXM suppresses *miRNA-155* expression in a dose-dependent manner (◇*P* < 0.05, versus 0 mg/kg group; ★ *P* < 0.05, versus 0.5 mg/kg group; △ *P* < 0.05, versus 2 mg/kg group; ◆ *P* < 0.05, versus 5 mg/kg group).

The expression of *miRNA-155* in the liver peaked at 12 hours after LPS exposure, approximately 70-fold (72.56 ± 9.34 )higher than the level observed in the NS group, and returned to the baseline level after 48 hours (Figure 4A).

### Effect of DXM on the expression of inflammatory cytokines and *miRNA-155* in liver tissue

Compared with the increased levels of TNF-α, IL-6, and IL-10 in liver tissues in the LPS group, those levels in the NS-LPS group were similar, whereas those in the DXM-LPS group were significantly decreased (Figure 2). Moreover, pretreatment with DXM appeared to prevent most of the pathological damages induced by LPS in the livers (Figure 3C). Compared with the increased expression of *miRNA-155* in the LPS and NS-LPS groups, the LPS-induced overexpression of *miRNA-155* was suppressed in the DXM-LPS group at 2 hours after treatment, and the greatest reduction occurred at 12 hours (Figure 4A). The expression level of *miRNA-155* in the DXM group was significantly down-regulated from 6 to 12 hours after treatment (Figure 4A). Furthermore, we found that DXM suppressed *miRNA-155* expression in a dose-dependent manner (Figure 4B).

## Discussion

To investigate the role of *miRNA-155* during sepsis, we detected the dynamic changes in *miRNA-155* expression in the liver of mice with LPS-induced sepsis and determined the regulatory effect of DXM on LPS-induced *miRNA-155* overexpression. We found that the expression of inflammatory mediators TNF-α, IL-6, and IL-10 significantly increased in the serum and liver after LPS exposure, which initiated the systemic inflammatory response and tissue injury. As a biomarker of liver injury, ALT expression increased at 2 hours following LPS challenge, and hepatocyte vacuolation, architectural distortion and nodular necrosis became evident over time. The expression of *miRNA-155* was also up-regulated, coincident with the up-regulation of inflammatory cytokines. These observations were consistent with our previous research [26]. 

The simultaneous increase in the expression of *miRNA-155* and inflammatory cytokines in mice with LPS-induced sepsis may attribute to their interactions. Previous study found that TNF-α stimulated the expression of *miRNA-155*[27]. On the other hand, *Eμ-miRNA-155* transgenic mice, which overexpress *miRNA-155* in cells from the B-cell lineage, produce more TNF-α when compared with wild-type mice after LPS exposure, indicating that *miRNA-155* may increase the production of inflammatory mediators [5]. Similarly, Jiang et al. [28] stimulated the breast cancer cells with IL-6 and found that expression of *miRNA-155* increase, suggesting that IL-6 also induces the *miRNA-155* expression. Compared with the pro-inflammatory cytokines TNF-α or IL-6, IL-10, which service as an important anti-inflammatory cytokine, prevents inflammation-mediated tissue damage by targeting various leukocytes and repressing excessive inﬂammatory responses [29]. It has been found to inhibit *miRNA-155* transcription by Toll-like receptors in a STAT3-dependent manner[30,31], and miRNA-155-deficient CD4+ T cells have been shown to produce more IL-10[32]. However, we observed simultaneous up-regulation of *miRNA-155* and IL-10. This may be related to the complex internal environment.

DXM can induce anti-inflammatory cytokines and inhibit pro-inflammatory cytokines at the transcriptional level, and thus could be used to treat sepsis. Consistent with previous studies, our data showed that the increased serum and tissue TNF-α and IL-6 levels in mice with early stages of sepsis were down-regulated by DXM. *miRNA-155* and DXM are both regulators of inflammation and immunity, but the relationship between them requires further investigation. Moschos et al [7] have found limited effect of DXM on LPS-induced expression of miRNAs in the lungs of mice. We found that the expression level of *miRNA-155* in the liver tissues from the LPS group was approximately 70 folds higher than the NS group, but this increase was impaired by DXM pretreatment. DXM may inhibit LPS-induced miRNA-155 expression indirectly by reducing the expression of pro-inflammatory cytokines. On other hand, the expression level of *miRNA-155* in the DXM group was even lower than that in the NS group. DXM reduced the basal expression level of miRNA-155 in mouse liver without LPS exposure, but did not reduce the basal expression of pro-inflammatory cytokines, indicating that other mechanisms may be involved in this process. In addition, we found that DXM suppressed *miRNA-155* expression in the liver tissues in a dose-dependent manner. This change in *miRNA-155* expression may be related to the levels of inflammatory cytokines. LPS may induce the expression of *miRNA-155* by promoting the expression of inflammatory factors. A wide variety of inflammatory cytokines, such as TNF-α and IL-6, can up-regulate *miRNA-155* expression. This up-regulation can be explained by the functions of pro-inflammatory transcription factors, such as NF-κB and AP-1, which promote the transcription of BIC genes. BIC genes contain the *pri-miRNA-155* transcription gene [33]. However, DXM may reduce the production of TNF-α by activating multiple signaling pathways, such as the PI3K, NF-κB, Akt/PKB, and MAPK signaling pathways [34,35]. Therefore, DXM may reduce *miRNA-155* expression in the liver by inhibiting pro-inflammatory cytokine expression. DXM reduced the baseline expression of *miRNA-155* but did not suppress the baseline levels of TNF-α and IL-6, suggesting that down-regulation of *miRNA-155* by DXM may not depend completely on the ability of DXM to suppress pro-inflammatory cytokines. It is likely that DXM may directly bind to the BIC genes to suppress *miRNA-155* expression. 

The regulation of *miRNA-155* by DXM may be a novel mechanism regulating inflammation and the immune response. Our results may provide the theoretical support for the application of DXM in the treatment of sepsis.
